# Assessment of *DAPK1* and *CAVIN3* Gene Promoter Methylation in Breast Invasive Ductal Carcinoma and Metastasis

**DOI:** 10.22074/cellj.2021.7251

**Published:** 2021-08-29

**Authors:** Esmat Ghalkhani, Mohammad Taghi Akbari, Pantea Izadi, Habibollah Mahmoodzadeh, Fatemeh Kamali

**Affiliations:** 1. Department of Medical Genetics, Faculty of Medical Sciences, Tarbiat Modares University, Tehran, Iran; 2. Department of Medical Genetics, School of Medicine, Tehran University of Medical Sciences, Tehran, Iran; 3. Department of Surgery, Cancer Institute of Iran, Tehran University of Medical Sciences, Tehran, Iran; 4. Iran National Tumor Bank, Cancer Institute of Iran, Tehran, University of Medical Sciences, Tehran, Iran

**Keywords:** Breast Cancer, *CAVIN3*, *DAPK1*, Metastasis, Methylation

## Abstract

**Objective:**

Metastasis might be latent or occur several years after primary tumor removal. Currently used methods for detection of distant metastasis have still some limitations. Blood tests may improve sensitivity and specificity of currently used screening procedures. The present study was designed to investigate promoter methylation status of *DAPK1* and *CAVIN3* genes in plasma circulating free DNA (cfDNA) samples in Iranian invasive ductal carcinoma (IDC) patients. We also investigated association of two gene promoter methylations with breast cancer (BC) and metastatic BC was also assessed.

**Materials and Methods:**

In this case-control study, MethySYBR assay was performed to determine *DAPK1* and *CAVIN3* promoter methylation status in breast IDC from 90 patients and 30 controls. Based on clinicopathological information, patient samples subdivided into stage I, II/III and IV groups (each group contained 30 individuals).

**Results:**

According to the results an increased promoter methylation level of the *DAPK1* gene in BC patients was observed. It was found that as disease progressed, the percentage of methylation was changed while it was not significant. Methylation changes in metastatic and non-metastatic BC revealed that methylation levels were significantly increased in metastatic than non-metastatic group. Analysis revealed that promoter methylation of *CAVIN3* gene in BC patients was significantly increased. The observed methylation changes from less to more invasive stages were not significant in the *CAVIN3* gene. Moreover, promoter methylation was changed in metastatic rather than non-metastatic condition, although it was not significant.

**Conclusion:**

Promoter hypermethylation of *DAPK1* and *CAVIN3* genes in plasma are associated with the risk of BC and they can be potential diagnostic biomarkers along with current methods. Additionally, association of aberrant *DAPK1* promoter methylation with metastasis suggests its potential usage as a non-invasive strategy for metastatic BC diagnosis.

## Introduction

Breast cancer (BC) is a heterogeneous and complex disease, as the most commonly detected cancer and the second cause of cancer mortality in women (1). The main cause death is due to metastasis to distant organs in BC patients (2). Some patients who display distant metastasis (stage IV disease) at the time of diagnosis are nearly incurable, only a minority of diagnosed BC patients belongs to this group. The rest of patients will also eventually experience distant metastasis (3). Metastasis early detection is necessary; this phenomenon might be latent or occur several years after primary tumor removal (4). 

Due to the problems with BC screening methods, identification and validation of non-invasive diagnostic biomarkers in the clinic seem necessary (5). Blood tests may improve sensitivity and specificity of currently used screening procedures (6). Alternatively, evaluation of tumor biomarkers as a non-invasive, accessible, easier and cost-effective approach could be valuable for early detection of BC (7). 

Different genetic and environmental factors are involved in BC and the role of DNA methylation needs to be identified (8). Gene methylation patterns in tumor tissues can indicate tumor invasion and recurrence (9). As tumors release DNA to the bloodstream, tumor methylation status can be evaluated by circulating tumor DNA (ctDNA) analysis non-invasively (10). Recently, the study of cell-free DNA (cfDNA) has attracted much attention as cancer biomarkers (11). As methylation alterations are among the early changes in tumorigenesis so, they are useful in early detection (12). In addition, it has been demonstrated that plasma methylation patterns can be used to accurately characterize cell type-specific cfDNA in disease and normal conditions (13).

Death-associated protein kinase 1 (DAPK1) is involved in cell cycle control, autophagy, apoptosis and tumor metastasis (14). It has been well established that *DAPK1* non-expressing cells were potentially more aggressive and metastatic due to the promoter hypermethylation (15). *DAPK1* hypermethylation has been identified in a wide range of tumors, such as kidney and bladder (16) as well as breast cancers (17, 18).

Caveolae Associated Protein 3 (CAVIN3), known as protein kinase C substrate, may participate in DNA repair pathway. It might impose apoptosis and cell cycle blockage, in addition to the suppression of tumor cell growth (19). Downregulation of *CAVIN3* was observed in the lung, ovarian, breast tissues (20) and its cell lines due to abnormal promoter hypermethylation (21).

The current study was designed to examine promoter methylation condition of *DAPK1* and *CAVIN3* genes in women diagnosed with breast IDC. Both genes are involved in the regulation of AKT (22, 23). Activation of AKT kinase is essential for many metastatic events, included escaping cells from tumor environment, activation of proliferation, suppression of apoptosis and activation of angiogenesis (24). These two genes are also involved in regulating p53 (25, 26). It has been shown that *TP53* gene is one of the potential regulators of metastasis and there is evidence that normal p53 regulates multiple steps of metastasis in a negative way (27). Here the promoter methylation condition was investigated for the first time in cfDNA plasma samples of Iranian invasive ductal carcinoma (IDC) patients. We aimed to evaluate the association of promoter methylation with breast cancer. We also decided to explore promoter methylation status of the mentioned genes in different BC stages and metastasis. Probable associations with clinicopathological parameters were also assessed.

## Materials and Methods

### Specimen collection

The present investigation was designed as a case-control study and included 90 patients with BC (age range of 30-66 years, mean ag=47 years) who were recruited from the Cancer Institute of Imam Khomeini Hospital (Tehran, Iran). All of the patients were diagnosed with breast IDC (stage I-IV) based on the tumor, nodes, and metastases (TNM) staging system. Inclusion criteria were primary diagnosed BC women who had received no chemotherapy/radiotherapy, with no previous history of BC or any other serious disease in them or their first-degree relatives. The clinicopathological information of patients was also taken. Thirty healthy women (age range of 30-55 years; mean age=44 years) were used in the control group. The controls with a history of cancer and other serious diseases in their first-degree relatives were excluded and specific factors such as smoking and alcohol consumption were considered. Written informed consent was also taken from all participants. The Ethics Review Committee of the Faculty of Medical Sciences of the Tarbiat Modares University (Tehran, Iran) approved the current study (IR.TMU.REC.1396.586). Approximately 5 ml peripheral blood was drawn from all individuals. 

### Plasma isolation and cfDNA extraction

Blood samples were immediately centrifuged at 1200 g for 15 minutes and the top plasma layer was centrifuged at 16000 g for 10 minutes. To check plasma hemolysis, the absorbance of plasma samples at 414 and 375 nm was measured and A414/A375 <2 was considered as hemolysis free plasma. Plasma cfDNA was isolated using the NucleoSpin® Plasma XS kit (Macherey-Nagel, Germany) based on the manufacturer’ s procedures with several modifications (28). 

### Sodium Bisulfite modification and DNA methylation investigation

cfDNA sodium bisulfite treatment was carried out as previously described by Yi et al. (29). Briefly, 50 µl freshly prepared 0.3 N NaOH (Merck, Germany) was added to cfDNA and incubated for 30 minutes in 37˚C. Then, 130 µl of 10 M (NH_4_) HSO_3_ -NaHSO_3_ bisulfite solution (Sigma-Aldrich, USA) was added and incubated for 30 minutes at 70˚C. The solution was afterward cooled at 4˚C. Gel/PCR Purification Mini Kit (YTA, Iran) was used to purify the cfDNA solution. Purified cfDNA was then mixed with 11 µl of fresh NaOH (0.2 N) followed by 10 minutes incubation in 37˚C. cfDNA was recovered by adding 150 µl of 4 M ammonium acetate (Merck, Germany), 3 µl glycogen and 750 µl cold absolute ethanol (Merck, Germany). The pellet was then eluted in µl of 10 mM Tris.HCl/1 mM EDTA (TE). 

The bisulfite-treated cfDNA was then used as a template for the MethySYBR method (30) to investigate the promoter methylation. In the present study, a two-step MethySYBR assay was used to enrich cfDNA using a pre-amplification step.

Real-time polymerase chain reaction (PCR) reactions were carried out in duplicate on StepOne™ Real-Time PCR System (Applied Biosystems, USA) in a total volume of 25 μl containing 0.5 μl from each primer (10 pM), 12 μl of 2X Real-Time PCR Master Mix (BIOFACT, South Korea), 9.5 μl RNase-free H_2_O and 2 μl cfDNA. Real-Time PCR conditions were as follows: initial denaturation at 95˚C for 10 minutes, 40 cycles of denaturation at 95˚C for 15 seconds, annealing at 56˚Cfor 30 seconds (*DAPK1* gene), 58˚C for 30 seconds (*CAVIN3* gene) and extension at 72˚C for 10 seconds. The following melt curve analysis was carried out: 95˚C for 15 seconds, 60˚C for 1 minute and 95˚C for 15 seconds. Fully methylated DNA was employed as a calibrator. To calculate the methylation percentage of each sample relative to fully methylated control, obtained “ΔΔC_t_ ” value (sample’s ΔC_t_ valu-calibrator’s ΔC_t_ value) entered into the 2^(−ΔΔCt) ^equation and then multiplied by 100. To amplify our target flanking region EXT-F1,2 and EXT-R primers were used. For amplification of the target region, M-F and M-R primers were employed. Primer sequences are as follows:

*DAPK1*:

EXT-F1: 5´-GTTAGGAATGTGGTTTTGGGG-3´

EXT-R: 5´-CCCTTT CTCTACACACATACCC-3´

EXT-F2: 5´-GAATGTGGTTTTGGGGATTGTTT-3´

M-F: 5´-CGGGGGTGTTATCGTTGTC-3´

M-R: 5´-GAAAAAATAAAACCCTCGCCCAAACG-3´

*CAVIN3*:

EXT-F1: 5´-TGAGTTATAGTTGGAGTTGGGGA-3´

EXT-R: 5´-TCCAACATAAAA ACCAACTTCTC-3´

EXT-F2: 5´-TAGTTGGAGTTGGGGAGGAGT-3´

M-F: 5´-TGTAGGTAG ACG GAGTAGAGC-3´

M-R: 5´-AACAAAATCACCACCGTCAC-3´

### Statistical analysis

SPSS (version 25) software (SPSS Inc., USA) was used to perform statistical analysis. The probable association of *DAPK1* and *CAVIN3* gene promoter methylations with breast cancer and metastatic BC were checked using an unpaired t test. Investigation of inter-group association was also performed using oneway ANOVA and Kruskal-Wallis one-way ANOVA (k samples). The results were interpreted significant if the P<0.05.

Diagnostic value, sensitivity and specificity of the *DAPK1* and *CAVIN3* promoter methylations in BC and metastatic BC were evaluated using receiver operator characteristic (ROC) curves. The area under the curve (AUC) with a 95% confidence interval (95% CI) was computed. 

## Results

### Clinicopathological features of patients

The clinicopathological characteristics of the 90 IDC patients involved in the study are summarized in Table 1. According to the age (<47/≥47), size (≤2/>2 cm) and lymph node condition (positive/negative) of the tumor, the patients were classified. 

### *DAPK1* promoter methylation status in breast cancer

**Fig.1:**
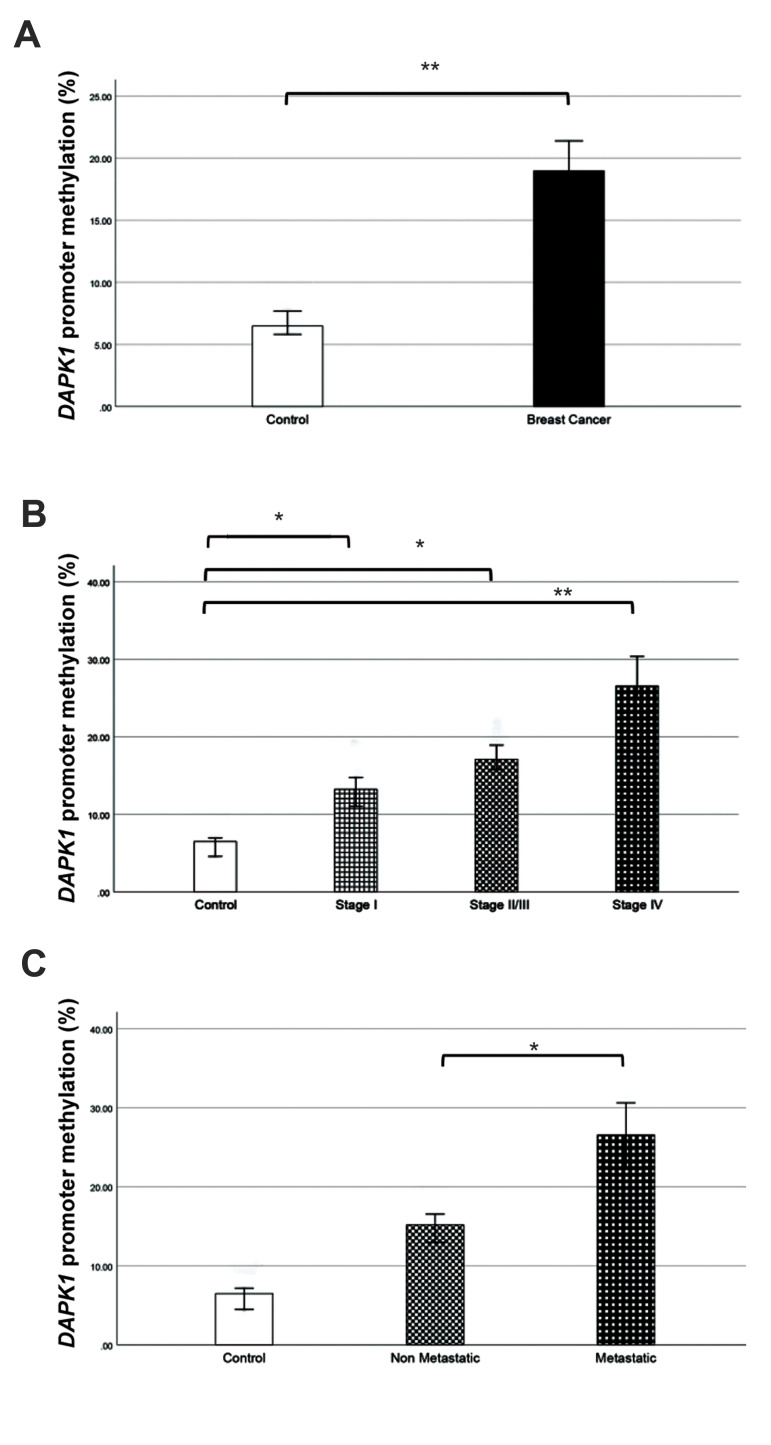
*DAPK1* methylation changes in BC. **A.** Changes in the *DAPK1* methylation levels of breast cancer patients compared to controls. The observed increase in methylation levels was significant. **B. ***DAPK1* promoter methylation levels in different stages of breast IDC. Methylation levels were increased as the disease was progressed. **C.** Methylation level changes in the metastatic and non-metastatic groups. It was found a significant increase in methylation levels in the metastatic group, rather than non-metastatic types. BC; Breast cancer, IDC; Invasive ductal carcinoma, *; P<0.05, and **; P<0.01.

In this study, MethySYBR method was used to quantify the promoter methylation percentage of genes. Association studies on the *DAPK1* gene promoter methylation level in BC were conducted. According to the results, 18.96% methylation was observed in BC patients compared to the control group (6.48%). The increased methylation level of *DAPK1* gene promoter was significant (P=0.001, Fig .1A). 

In order to investigate promoter methylation status in IDC tumor progression, *DAPK1* gene promoter methylation was assessed in stages I, II/III and IV. Methylation percentage in the stages I, II/III and IV were 13.23%, 17.10% and 26.54, respectively. Regarding the evaluated results, the observed differences between control group and all of the stages were significant. Stage I compared to the control group showed increased promoter methylation (P=0.046). Similar results were also observed in stage II/III (P=0.036) and stage IV (P=0.002) rather than the control group. When the different groups were compared, it was found that methylation level was increased from stage I to stage II/III (P=0.493) and increased methylation was also observed in the progression from stage II/III to stage IV (P=0.155). Although inter-group comparison did not show statistically significant results, except for the increased methylation level from stage I to stage IV (P=0.046, Fig .1B).

**Table 1:** Breast invasive ductal carcinoma patient’s clinicopathological features


Variable			n (%)

Age (Y)			
	<47		44 (48.88)
	≥47		46 (51.11)
T stage			
	T1 ≤2		38 (42.22)
	T2	}>2	15 (16.67)
	T3		19 (21.11)
	T4		18 (20)
Lymph node involvement			
	Positive		54 (60)
	Negative		36 (40)
Metastasis status			
	Metastatic		30 (33.33)
	Non-metastatic		60 (66.67)
Clinical TNM staging			
	I		30 (33.33)
	II and III		30 (33.33)
	IV		30 (33.33)


TNM; Tumor, nodes, and metastases.

### *DAPK1* gene promoter methylation and breast cancer metastasis

To check the association of *DAPK1* gene promoter methylation levels with BC metastasis, methylation changes were assessed in stages I, II/III (as non-metastatic group) and IV (as metastatic group). Results revealed that methylation levels were significantly increased in metastatic (26.54%) than non-metastatic group (15.17%, P=0.016). According to these results, it was determined that methylation level of *DAPK1* gene promoter was associated with BC metastasis (Fig .1C).

### *CAVIN3* gene promoter methylation status in breast cancer

**Fig.2:**
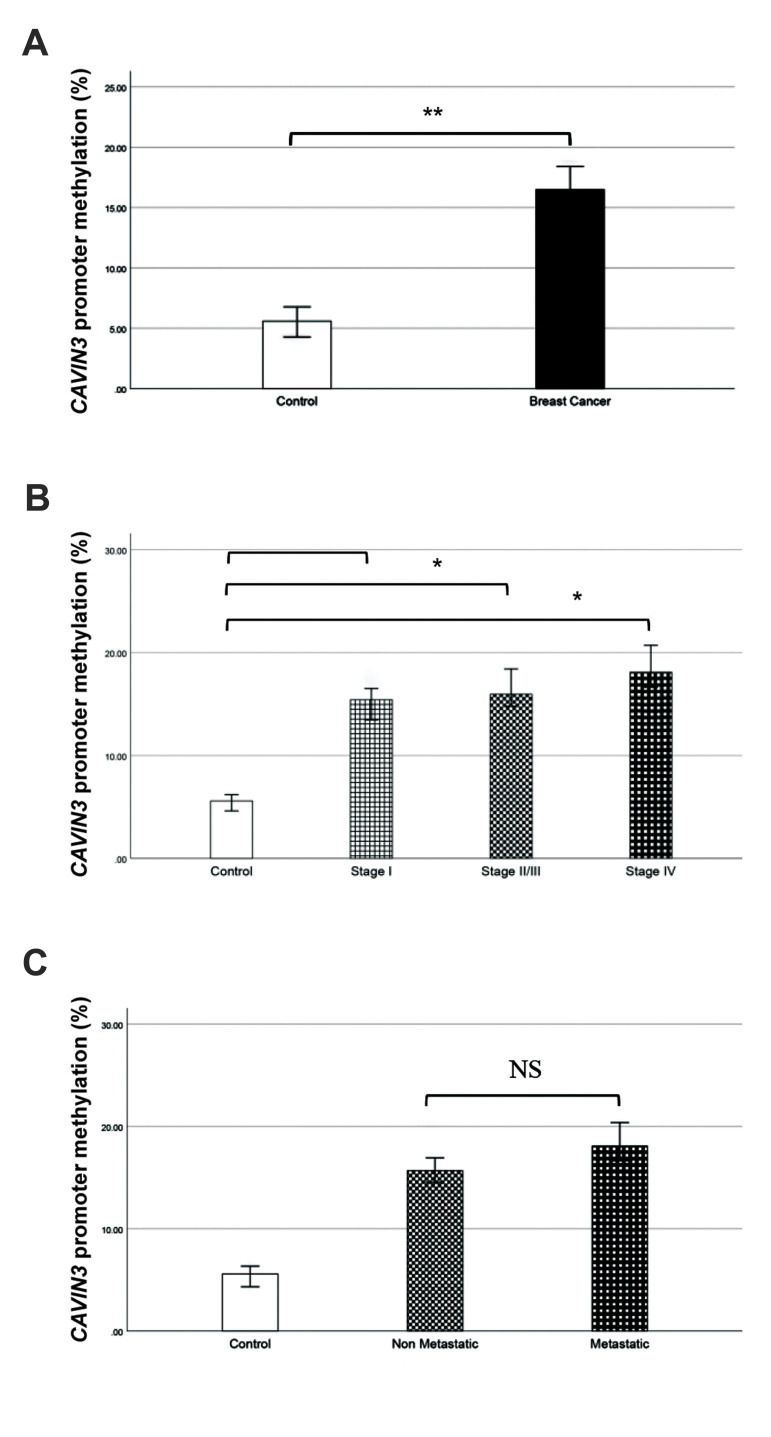
*CAVIN3* methylation changes in BC. **A.** Promoter methylation of *CAVIN3* gene in breast cancer patients versus control. Methylation levels were increased significantly in breast cancer patients compared to the control group. **B.** Promoter methylation of *CAVIN3* gene in different stages of breast cancer rather than control, methylation changes from less to more invasive stages were negligible. **C.** *CAVIN3* promoter methylation in metastatic and non-metastatic breast cancer, methylation changes between metastatic and non-metastatic groups were not significant. BC; BC; Breast cancer, *; P<0.05, **; P<0.01, and NS; Non-significant.

Data analysis revealed that promoter methylation level of *CAVIN3* gene was significantly increased in BC patients (16.49%) rather than normal individuals (5.58%, P=0.002, Fig .2A).

Promoter methylation of the studied gene was also increased significantly in IDC stage I (15.42%) versus control, stage II/III (15.95%) compared to control as well as stage IV (18.09%) rather than the control group, with respectively 0.025, 0.022 and 0.019 P values. To evaluate association of promoter methylation of the *CAVIN3* gene with breast IDC progression, an inter-group comparison was also performed. Enhanced promoter methylation was observed from stage I to stage II/III, although it was not statistically significant (P=0.740). A non-significant methylation enhancement was also observed in stage II/III compared to stage IV (P=0.678). It was revealed that promoter methylation in this gene was increased from stage I to stage IV, although this observation was not statistically significant (P=0.092, Fig .2B).

### *CAVIN3* gene promoter methylation and breast cancer metastasis

Promoter methylation of *CAVIN3* gene was increased in metastatic condition (stage IV, 18.09%) rather than non-metastatic condition (stages II/III, 15.68%), although it was not statistically significant (P=0.678, Fig .2C).

### Association of *DAPK1* and *CAVIN3* gene promoter methylation changes with clinicopathological parameters

As it was mentioned previously, the studied samples were classified in different groups according to age (<47/≥47 years old), size (≤2/>2 cm) and lymph node condition (positive/ negative) of the tumor. Promoter methylation was then investigated in each group. There was no significant association between *DAPK1* and *CAVIN3* gene promoter methylation and age, tumor size and lymph node status. The obtained results are summarized in Table 2.

**Table 2:** Association of *DAPK1* and *CAVIN3* gene promoter methylations with clinicopathological parameters


Clinicopathological parameters		Number of cases	*DAPK1* (P value)	*CAVIN3* (P value)

Age (Y)			0.68	0.62
	<47	44		
	≥47	46		
Tumor size (cm)			0.30	0.93
	≤2	38		
	>2	52		
Lymph node involvement			0.056	0.63
	Positive	54		
	Negative	36		


### Diagnostic value of *DAPK1* and *CAVIN3* gene promoter methylations in breast cancer

**Fig.3:**
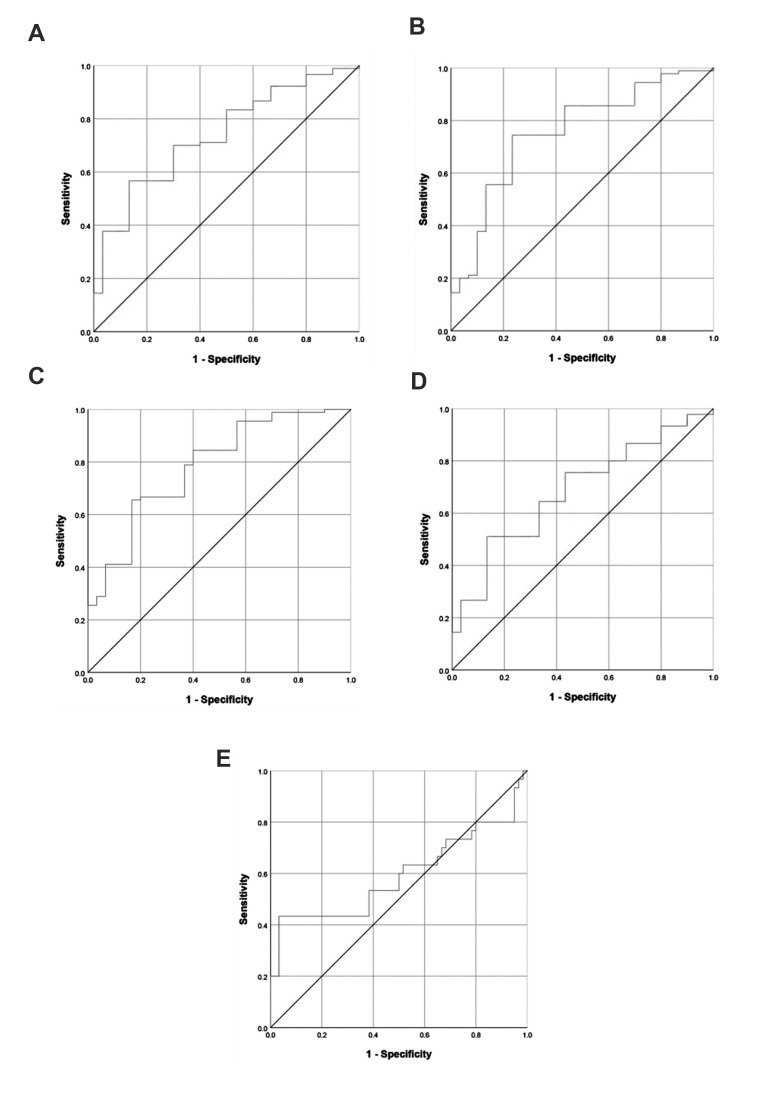
ROC curve analysis of *DAPK1* and *CAVIN3* genes promoter methylation in BC. ROC curves were constructed to assess diagnostic potential of **A.** *DAPK1*, **B.** *CAVIN3* promoter methylation and** C. **Their combination in BC discrimination. **A.** The AUC was 0.732 (P<0.001). **B.** The AUC was 0.740 (P=0.00). **C. **Using *DAPK1* and *CAVIN3* genes combination, the AUC reached 0.799 (P=0.00). ROC curves were also generated to determine the efficiency of **D. ***DAPK1* and **E.** *CAVIN3* promoter methylation in BC metastasis diagnosis. **D.** The AUC was 0.692 (P=0.002). **E. **The AUC was 0.584 (P=0.193). ROC; Receiver operator characteristic, BC; Breast cancer, and AUC; Area under the curve.

Using ROC curves, diagnostic accuracy of *DAPK1* and *CAVIN3* gene promoter methylations in discriminating BC was determined. According to the evaluated results, *DAPK1* gene promoter hypermethylation was able to distinguish the BC patients from the control group and showed a sensitivity of 70% and specificity of 66.7% with an AUC of 0.732 (95% CI=0.633-0.831, P=0.00). *CAVIN3* gene promoter hypermethylation achieved an AUC of 0.740 (95% CI=0.638-0.843, P=0.00) with a sensitivity of 70% and specificity of 70%. Combination of the two genes improved diagnostic value and reached an AUC of 0.799 (95% CI=0.707-0.891, P=0.00) with respectively sensitivity and specificity of 71.1% and 73.3% (Fig .3A-C). The obtained results were in accordance with our MethSYBR data.

### Diagnostic value of *DAPK1* and *CAVIN3* gene promoter methylations in breast cancer metastasis

ROC curve analysis was used to determine diagnostic potential of *DAPK1* and *CAVIN3* gene promoter methylations in differentiating BC metastasis. The discriminatory performance of the two evaluated genes differed significantly. *DAPK1* gene promoter hypermethylation demonstrated an AUC of 0.692 (95% CI=0.591-0.793, P=0.002) with 64.4% sensitivity and 66.7% specificity, while *CAVIN3* gene promoter hypermethylation was not able to distinguish metastatic BC patients from non-metastatic types. In *CAVIN3* gene, AUC was 0.584 (95% CI=0.444-0.724, P=0.193) with respective sensitivity and specificity of 53.3% and 61.7% (Fig .3D, E).

## Discussion

In the present study, association *DAPK1*and *CAVIN3* gene promoter methylations BC and BC metastasis was explored. In this study, promoter methylation status of *DAPK1* and *CAVIN3* genes were examined in women diagnosed with breast IDC. Here the promoter methylation condition was investigated for the first time in cfDNA plasma samples of Iranian IDC patients. We aimed to evaluate association of gene promoter methylation with breast cancer. We also decided to explore promoter methylation of the mentioned genes in different BC stages and metastasis. Probable associations with clinicopathological parameters were also assessed.

It was found that promoter methylation of *DAPK1* gene in BC patients was significantly increased. Studies conducted by Tserga et al. (31) and Cho et al. (32) showed that *DAPK1* gene promoter was respectively methylated 37.5% and 14.1% in breast primary tumors. Spitzwieser et al. (33) observed 62% *DAPK1* gene promoter methylation in invasive ductal and lobular carcinoma. Different results observed in various studies may be due to different factors such as sample size, race, treatment status, nutritional status and family history (34). 

In the present study, it was observed that level of *DAPK1* gene promoter methylation in plasma samples of patients with IDC was 18.96%. Our findings are in agreement with these studies, as they showed that promoter methylation level of *DAPK1* gene was increased in BC compared to normal individuals. Inactivation of *DAPK1* gene expression due to hypermethylation has been frequently found in a variety of cancers and it was associated with tumor invasiveness (35, 36). It has been proved that *DAPK1* plays tumor suppressor kinase role (37) which is in accordance with our obtained results. Increasing promoter methylation might lead to the downregulation of this gene, which plays an important role in tumorigenesis. ROC curve analysis revealed that *DAPK1* gene promoter methylation could successfully be used as a potential biomarker in BC diagnosis. This test interestingly approved the associations that we assessed.

It was also observed that there is a significant increase in the methylation level of *DAPK1* gene in all of the stages (I, II/III and IV) compared to the control group. Regarding the association between promoter methylation of this gene and progression of breast cancer, an increased methylation level from less invasive stages to more aggressive stages was detected, but they were not significant. Although, in a study done by Yadav et al. (34) an increased methylation level was found by progressing the disease. An expected increasing trend was observed from less to more invasive stages in our study, which is in accordance with previous experiments. Obtaining non-significant results might be attributed to the small number of samples. 

In our study, considering the metastatic BC patients and non-metastatic ones, a significant increase of methylation level was observed in the metastatic group. Botezatu et al. (38) showed 64% *DAPK1* promoter methylation in advanced stages of IDC. In the current study, promoter methylation level of the *DAPK1* gene was 26.54% in the metastatic group and it was associated with BC metastasis. Our data are completely consistent with the mentioned study. We suggest that promoter hypermethylation of *DAPK1* gene may lead to its downregulation and this condition might be one of the effective factors in BC metastasis. Given the data obtained from ROC assessment, it can be concluded that promoter methylation status of *DAPK1* can be served as a potential biomarker for BC metastasis detection. The results of our study about *DAPK1* promoter methylation are consistent with ROC findings. In the clinicopathological survey, no significant association was observed between promoter methylation condition of the *DAPK1* gene and any of the clinicopathological factors. Although in Yadav et al. (34) study, a relation between the promoter methylation condition of *DAPK1* gene and clinicopathological features was found in BC patients. This inconsistent finding might be associated with the small number of studied samples, which further needs to be invested.

In the present study, significantly increased promoter methylation in *CAVIN3* gene (16.49%) was observed in plasma samples of BC patients rather than control individuals. In the present study obtained results are consistent with a study performed by Li et al. (39) who found an increased methylation level in BC tissues. CAVIN3 is a tumor suppressor protein (20) whose methylation alterations were associated with some tumor types, such as breast tumors (21). In this study, we suggested that *CAVIN3* gene might play as a tumor suppressor gene as it shows enhanced promoter methylation. In a study conducted by Xu et al. (21) it was found that downregulation of *CAVIN3* gene was associated with hypermethylation of promoter CpG dinucleotides (60% methylation level) in primary breast tumors. It can be concluded that hypermethylation of *CAVIN3* promoter can down-regulate this gene which may eventually lead to BC tumorigenesis. With respect to ROC analysis, the promoter methylation of the *CAVIN3* gene is able to be used, as a possible diagnostic biomarker in breast cancer. This is in accordance with the association study. Combination of the *DAPK1* and *CAVIN3* gene promoter methylations showed better results in the diagnosis of breast cancer.

In our study, investigation of *CAVIN3* promoter methylation levels also revealed a significant increase in all of the stages compared to the control group. In addition, different stage alterations proved an increased methylation level in progressing from less to more invasive stages, but these changes could not reach the significance threshold. Increased promoter methylation of *CAVIN3* from less to more invasive stages might cause a reducing trend in *CAVIN3* expressions. Therefore, we can suggest that enhancement of promoter methylation may be effective in BC progression. Obtaining a non-significant result might be associated with sample size, which requires more investigations.

Furthermore, in the metastatic group, increased *CAVIN3* promoter methylation level was not significant. Although in a study performed by Li et al. (40) *CAVIN3* gene methylation was informative for predicting metastatic breast cancer. We suggest investigating association in a larger population, since non-significant results in the present study might be associated with the small size of samples. ROC analysis of *CAVIN3* gene promoter methylation did not reach a significant threshold. No significant association between *CAVIN3* gene promoter methylation changes and clinicopathological characteristics was also observed.

## Conclusion

Overall, the obtained results should be interpreted cautiously and it seems necessary to confirm them in other independent studies. In conclusion, promoter hypermethylation of *DAPK1* and *CAVIN3* genes in plasma are associated with the risk of BC and they can be potential diagnostic biomarkers proposed for the first time in the Iranian population along with the current methods. In addition, aberrant *DAPK1* promoter methylationpositively associates with metastasis of breast cancer. It suggests the potential usage of promoter methylation as a non-invasive strategy for metastatic BC diagnosis. Further analysis of these genes could be helpful to reveal their potential roles in BC development and metastasis. 
